# Increased motor cortex inhibition as a marker of compensation to chronic pain in knee osteoarthritis

**DOI:** 10.1038/s41598-021-03281-0

**Published:** 2021-12-14

**Authors:** Marcel Simis, Marta Imamura, Paulo S. de Melo, Anna Marduy, Kevin Pacheco-Barrios, Paulo E. P. Teixeira, Linamara Battistella, Felipe Fregni

**Affiliations:** 1 Hospital das Clinicas HCFMUSP, Faculdade de Medicina, Universidade de São Paulo, São Paulo, Brazil; 2 Neuromodulation Center and Center for Clinical Research Learning, Spaulding Rehabilitation Hospital and Massachusetts General Hospital, Harvard Medical School, 96 13th Street, Charlestown, Boston, MA USA; 3 Universidad San Ignacio de Loyola, Vicerrectorado de Investigación, Unidad de Investigación Para la Generación y Síntesis de Evidencias en Salud, Lima, Peru

**Keywords:** Biomarkers, Neurology, Osteoarthritis

## Abstract

This study aims to investigate the associative and multivariate relationship between different sociodemographic and clinical variables with cortical excitability as indexed by transcranial magnetic stimulation (TMS) markers in subjects with chronic pain caused by knee osteoarthritis (OA). This was a cross-sectional study. Sociodemographic and clinical data were extracted from 107 knee OA subjects. To identify associated factors, we performed independent univariate and multivariate regression models per TMS markers: motor threshold (MT), motor evoked potential (MEP), short intracortical inhibition (SICI), intracortical facilitation (ICF), and cortical silent period (CSP). In our multivariate models, the two markers of intracortical inhibition, SICI and CSP, had a similar signature. SICI was associated with age (β: 0.01), WOMAC pain (β: 0.023), OA severity (as indexed by Kellgren–Lawrence Classification) (β: − 0.07), and anxiety (β: − 0.015). Similarly, CSP was associated with age (β: − 0.929), OA severity (β: 6.755), and cognition (as indexed by the Montreal Cognitive Assessment) (β: − 2.106). ICF and MT showed distinct signatures from SICI and CSP. ICF was associated with pain measured through the Visual Analogue Scale (β: − 0.094) and WOMAC (β: 0.062), and anxiety (β: − 0.039). Likewise, MT was associated with WOMAC (β: 1.029) and VAS (β: − 2.003) pain scales, anxiety (β: − 0.813), and age (β: − 0.306). These associations showed the fundamental role of intracortical inhibition as a marker of adaptation to chronic pain. Subjects with higher intracortical inhibition (likely subjects with more compensation) are younger, have greater cartilage degeneration (as seen by radiographic severity), and have less pain in WOMAC scale. While it does seem that ICF and MT may indicate a more acute marker of adaptation, such as that higher ICF and MT in the motor cortex is associated with lesser pain and anxiety.

## Introduction

Chronic pain is one of the main causes of disability worldwide^1^ and a major burden of knee osteoarthritis (OA), causing impaired function and decreased quality of life^2^. The chronic pain caused by OA sometimes is not correlated with the severity of the peripheral injury. Neuroplastic changes in pain-related circuits, resulting in maladaptive neuroplasticity, lead to a perpetuation of pain^3,4^. This process has been shown in other chronic pain conditions such as phantom limb pain, fibromyalgia, and low back pain, supporting the idea of a central nervous system modulation of chronic pain^5,6^. In these cases, central sensitization of nociceptive pathways may be present, which, along with an impairment in the descending pain modulation pathway, plays an essential role in how patients perceive pain in the long term^2,7^. These modifications on the central nervous system imply an imbalance of the neural stability between excitability and inhibition^8^. Current evidence has shown that a deficit in neuronal inhibition occurs in patients with motor disability and pain conditions^9–11^ and this lack of cortex inhibition is associated with more disability^12^. In fact, one of the contributors of pain in these patients may be an impaired cortical inhibitory system that cannot compensate for excessive peripheral increased nociception.

The specific mechanisms involved in excitability-inhibition balance and brain plasticity regulation are not completely understood. In these circumstances, transcranial magnetic stimulation is a commonly used and feasible tool to measure the functional level of motor cortex excitability^13–15^ through markers such as resting motor threshold (rMT), the intensity of a stimulus necessary to produce a motor evoked potential (MEP) which is the electrical signal sent through descending neural pathways when the motor cortex is stimulated, both indirect markers of corticospinal excitability; cortical silent period (CSP), which represents momentary suppression of MEP due to GABAergic inhibition; and short intracortical inhibition (SICI) and intracortical facilitation (ICF), which represent inhibitory and excitatory activation of interneurons within the motor cortex^16,17^. A systematic review on TMS finding in chronic pain patients reported reduced SP and SICI; however, most of the included studies were underpowered (less than 30 participants), included heterogenous populations, and did not assess which clinical variables are associated with this reduced cortical inhibition^18^.

Our main hypothesis is that clinical characteristics in OA subjects, such as pain, motor function, and cognitive-emotional behavior, are associated with the quantity of intracortical inhibition (and other parameters of excitability). For this reason, the objective of this study is to investigate the association of different sociodemographic and clinical variables and the cortex excitability conveyed by MT, MEP, CSP, SICI, and ICF TMS markers in subjects with chronic pain caused by knee OA. Considering that the central mechanisms of chronic pain are important in knee OA, the cortical excitability measures (as assessed by TMS) could indicate the neuroplastic alterations in this condition^21^. Therefore, understanding their association with clinical and demographic factors can possibly contribute to the identification of specific OA populations with less or more maladaptive plasticity (who might chronify pain easily). Knowing these associated factors is the starting point for phenotype chronic pain associated with knee OA and thus develop more precise treatments.

## Results

Table 1 summarizes the descriptive characteristics of the study sample. Pain is reported as moderate in both knees and patients present with low cognitive, anxiety, and depression scores.

**Table 1:** Clinical and sociodemographic characteristics of knee OA study participants.

Variables	All knee OA subjects (N = 107)
**Demographics**	
Age	67.78 ± 9.57
Gender (%)	
Male	18 (16.82)
Female	89 (83.18)
Time of ongoing pain (months)	95.50 ± 99.19
Weight (kilograms)	80.01 ± 15.62
Height (meters)	1.58 ± 0.09
BMI	31.97 ± 5.33
**Functional assessments**	
Kellgren–Lawrence classification	
Right	2.50 ± 1.19
Left	2.33 ± 1.17
Average	2.39 ± 1.14
Total knee arthroplasty (%)	
Right	3 (2.80)
Left	3 (2.80)
Ten-minute walking test	11.86 ± 6.93
Six-minute walking test (distance in meters)	307.19 ± 109.26
Timed up-and-go	15.66 ± 7.70
Berg balance scale	48.27 ± 8.27
Epworth sleepiness scale	10.39 ± 5.87
Medical research council scale bilateral average	4.21 ± 0.60
WOMAC stiffness	4.62 ± 2.00
WOMAC physical function	36.13 ± 13.89
WOMAC pain	10.98 ± 3.97
**Pain assessments**	
Bilateral pain (%)	106 (99.06)
Pain pressure threshold (kPa)	
Knee	
Right	4.87 ± 2.57
Left	4.79 ± 2.49
Average	4.82 ± 2.49
Upper Limb	
Right	5.84 ± 2.06
Left	5.56 ± 2.13
Average	5.70 ± 2.02
Conditioned pain modulation (kPa)	− 1.02 ± 1.29
Visual analogue scale	
Right	5.70 ± 2.02
Left	5.40 ± 2.80
Average	5.55 ± 2.06
Pain catastrophizing scale	14.49 ± 11.01
**Cognitive/emotional assessments**	
HAM-D scale	9.37 ± 5.55
HAD	
Anxiety	5.92 ± 4.23
Depression	4.27 ± 3.56
Montreal cognitive assessment	
Education Level (years)	9.72 ± 3.71
Total score	21.02 ± 4.95
**Neurophysiological measures**	
Short-interval Intracortical Inhibition (SICI)	
Right	0.46 ± 0.32
Left	0.49 ± 0.32
Bilateral	0.47 ± 0.27
Intracortical facilitation (ICF)	
Right	1.60 ± 0.65
Left	1.70 ± 0.82
Bilateral	1.64 ± 0.57
Cortical silent period (CSP) (ms)	
Right	91.83 ± 35.82
Left	80.81 ± 31.67
Bilateral	86.32 ± 31.46
Motor threshold (MT) (µV)	
Right	52.73 ± 11.59
Left	50.97 ± 11.00
Bilateral	51.36 ± 11.45
Motor evoked potential (MEP) (mV)	
Right	1.75 ± 1.30
Left	1.87 ± 2.02
Bilateral	1.81 ± 1.41

*BMI* Body Mass Index, *WOMAC Pain/Stiffness/Function* Western Ontario and MacMaster Universities Osteoarthritis Index Pain/Stiffness/Function section, *HAM-D* Hamilton Depression Rating Scale, *HAD-Anxiety* Hospital Anxiety and Depression Scale—Anxiety section.

### Short intracortical inhibition (SICI)

Short Intracortical Inhibition conveyed some significant associated variables in its univariate analysis including age (p = 0.039), gender (p = 0.074), and Berg Balance Scale results (p = 0.053) (Table 2). From our multivariate model, age is depicted as a positive associated variable to SICI (β: 0.011, 95% CI 0.003 to 0.017; p = 0.004); as patients get older, they have less cortical inhibition (as indexed by SICI). Similarly, the WOMAC pain scale scores also portrayed a positive correlation with SICI (β 0.024, 95% CI 0.008 to 0.041; p = 0.004), indicating that the more pain a patient feels, less SICI occurs. A significant negative correlation was found between the Kellgren-Lawrence classification and SICI (β − 0.070, 95% CI − 0.12 to − 0.017; p = 0.001). Given a positive relationship between OA severity and SICI, the more severe K–L classification, larger cortical inhibition. HADS-anxiety also demonstrated a small inverse correlation (β − 0.015, 95% CI − 0.030 to 0.0003, p = 0.046). Moreover, other variables were confounders, such as BMI and Conditioned Pain Modulation (CPM) (Table 2).

**Table 2:** Univariate analysis of clinical and sociodemographic variables associated with SICI and final model of multivariate analysis with significant associated variables with SICI.

Variable	β-coefficient	95% CI	Unadjusted p-value	
Age	0.006	0.0004 to 0.011	0.035	
Gender	− 0.126	− 0.26 to 0.012	0.074	
Time of ongoing pain (months)	0.0001	− 0.0004 to 0.0006	0.709	
Weight (kg)	0.0007	− 0.002 to 0.004	0.688	
Height (m)	0.286	− 0.27 to 0.847	0.313	
BMI	− 0.0002	− 0.01 to 0.010	0.966	
**Kellgren–Lawrence classification**				
Right	− 0.024	− 0.069 to 0.021	0.300	
Left	− 0.017	− 0.064 to 0.029	0.456	
Average total	− 0.021	− 0.070 to 0.027	0.387	
**Knee arthroplasty**				
Right	0.018	− 0.372 to 0.408	0.928	
Left	− 0.120	− 0.440 to 0.190	0.439	
Ten-minute walking test	0.003	− 0.005 to 0.010	0.469	
Six-minute walking test (distance in meters)	− 0.0002	− 0.0008 to 0.0002	0.324	
Timed up-and-go	0.003	− 0.004 to 0.010	0.390	
Berg Balance scale	− 0.006	− 0.018 to 0.0001	0.053	
Epworth sleepiness scale	0.001	− 0.008 to 0.010	0.756	
Medical research council scale average	− 0.040	− 0.126 to 0.048	0.370	
WOMAC stiffness	− 0.001	− 0.027 to 0.025	0.952	
WOMAC physical function	0.002	− 0.002 to 0.006	0.301	
WOMAC pain	0.007	− .007 to 0.020	0.322	
**Pain pressure threshold (kPa)**				
Knee				
Right	0.0007	− 0.020 to 0.021	0.948	
Left	0.006	− 0.015 to 0.027	0.582	
Bilateral	0.003	− 0.018 to 0.024	0.758	
Upper limb				
Right	0.002	− 0.023 to 0.028	0.859	
Left	− 0.004	− 0.029 to 0.021	0.738	
Bilateral	− 0.001	− 0.027 to 0.025	0.927	
Conditioned pain modulation (kPa)	− 0.002	− 0.050 to 0.046	0.930	
**Visual analogue scale**				
Right	0.003	− 0.015 to 0.022	0.733	
Left	0.014	− 0.044 to 0.033	0.130	
Bilateral	0.016	− 0.010 to 0.041	0.207	
Pain catastrophizing scale	− 0.0003	− 0.005 to 0.005	0.907	
HAM-D scale	0.0004	− 0.009 to 0.010	0.945	
**Hospital anxiety and depression scale**				
Anxiety	− 0.010	− 0.022 to 0.003	0.118	
Depression	0.004	− 0.010 to 0.019	0.562	
**Montreal cognitive assessment**				
Education Level (years)	− 0.005	− 0.019 to 0.001	0.520	
Total score	− 0.001	− 0.011 to 0.010	0.898	

*Model 2.* multivariate analysis for SICI			Adjusted p-value	R-squared: 0.2862
Age	0.011	0.003 to 0.017	0.004	
Gender	− 0.13	− 0.27 to 0.010	0.069	
BMI	0.006	− 0.005 to 0.017	0.272	
Kellgren Lawrence classification	− 0.070	− 0.12 to − 0.017	0.001	
Conditioned pain modulation	− 0.023	− 0.074 to 0.021	0.311	
WOMAC pain	0.024	0.008 to 0.041	0.004	
HAD-anxiety	− 0.015	− 0.030 to 0.0003	0.046	

*BMI* Body Mass Index, *WOMAC Pain/Stiffness/Function* Western Ontario and MacMaster Universities Osteoarthritis Index Pain/Stiffness/Function section, *HAM-D* Hamilton Depression Rating Scale, *HAD-Anxiety* Hospital Anxiety and Depression Scale—Anxiety section.

### Cortex silent period (CSP)

Possible relevant associated variables with CSP in knee OA chronic pain in the univariate analysis were BMI (p = 0.008), left knee Kellgren–Lawrence severity classification (p = 0.075), total arthroplasty (p = 0.026), right knee visual analogue pain scale score (p = 0.014), and total MoCA cognitive assessment scores (p = 0.079) (Table 3). In our multivariate model, we found a strong, positive correlation between the KL average score for both knees was identified (β: 6.755, 95% CI: 0.662 to 12.848; p = 0.030), implying that the more severe OA patients have, the higher their silent period amplitude (therefore, higher intracortical inhibition). Moreover, negative correlations between CSP and age (β: − 0.929, 95% CI − 1.678 to − 0.181; p = 0.016) and MoCA scores (β: − 2.106, 95% CI − 3.64 to − 0.568; p = 0.008) indicate inverse linear relationships meaning that as a patient gets older, their SP is diminished, and the higher cognitive function these patients have, the less CSP they depict. Education level was included in the model as it is a significant confounder given its relationship to MoCA (Table 3).

**Table 3:** Univariate analysis of clinical and sociodemographic variables associated with CSP and final model of multivariate analysis with significant associated variables with CSP.

Variables	β-coefficient	95% CI	Unadjusted p-value	
Age	− 0.488	− 1.117 to 0.14	0.127	
Gender	0.390	− 15.80 to 16.59	0.962	
Time of ongoing pain (months)	0.00647	− 0.055 to 0.068	0.836	
Weight (kg)	0.296	− 0.088 to 0.68	0.13	
Height (m)	− 28.001	− 92.556 to 36.555	0.392	
BMI	1.510	0.408 to 2.618	0.008	
**Kellgren–Lawrence classification**				
Right	1.880	− 3.25 to 7.01	0.469	
Left	4.690	− 0.477 to 9.859	0.075	
Average total	4.089	− 1.26 to 9.44	0.133	
**Knee arthroplasty**				
Right	27.160	− 17.49 to 71.81	0.23	
Left	− 40.940	− 76.966 to − 4.92	0.026	
Ten-minute walking test	0.580	− 0.288 to 1.45	0.188	
Six-minute walking test (distance in m)	− 0.036	− 0.091 to 0.019	0.199	
Timed up-and-go	0.136	− 0.65 to 0.92	0.731	
Berg balance scale	− 0.567	− 1.29 to 0.159	0.125	
Epworth sleepiness scale	1.268	0.26 to 2.27	0.014	
Medical research council scale average	− 6.508	− 16.507 to 3.49	0.2	
WOMAC stiffness	− 0.035	− 3.078 to 3.0074	0.982	
WOMAC physical function	0.270	− 0.16 to 0.70	0.217	
WOMAC pain	1.237	− 0.27 to 2.749	0.108	
**Pain pressure threshold (kPa)**				
Knee				
Right	0.174	− 2.194 to 2.541	0.885	
Left	− 0.046	− 2.493 to 2.402	0.971	
Bilateral	0.070	− 2.378 to 2.518	0.955	
Upper Limb				
Right	− 0.326	− 3.277 to 2.62	0.827	
Left	− 0.420	− 2.898 to 2.81	0.977	
Bilateral	− 0.190	− 3.199 to 2.81	0.899	
Conditioned pain modulation (kPa)	2.889	− 2.297 to 8.077	0.271	
**Visual analogue scale**				
Right	1.880	− 0.23 to 3.99	0.08	
Left	0.640	− 1.52 to 2.81	0.558	
Bilateral	2.369	− 0.54 to 5.28	0.11	
Pain Catastrophizing Scale	0.345	− 0.21 to 0.90	0.223	
HAM-D Scale	0.789	− 0.308 to 1.886	0.157	
**Hospital Anxiety and Depression Scale**				
Anxiety	0.900	− 0.53 to 2.34	0.216	
Depression	0.073	− 1.65 to 1.798	0.933	
**Montreal cognitive assessment**				
Education Level (years)	− 0.529	2.206 to 1.147	0.533	
Total score	− 1.099	− 2.328 to 0.13	0.079	

*Model 4.* Multivariate Analysis for CSP (ms)			Adjusted p-value	R-Squared: 0.2120
Age	− 0.929	− 1.678 to -0.181	0.016	
Education	4.907	− 4.580 to 14.395	0.306	
Kellgren Lawrence classification Average	6.755	0.662 to 12.848	0.030	
MoCA total	− 2.106	− 3.64 to -0.568	0.008	
HAD-anxiety	− 0.434	− 2.118 to 1.250	0.609	

*BMI* Body Mass Index, *WOMAC Pain/Stiffness/Function* Western Ontario and MacMaster Universities Osteoarthritis Index Pain/Stiffness/Function section, *HAM-D* Hamilton Depression Rating Scale, *HAD-Anxiety* Hospital Anxiety and Depression Scale-Anxiety section.

### Intracortical facilitation (ICF)

Univariate analysis of associated factors to intracortical facilitation (ICF) conveyed significance for time of ongoing pain (p = 0.062), the visual analogue scale pain score (p = 0.03) and the anxiety subscale of the Hospital Anxiety and Depression Scale (HADS) (p = 0.044) (Table 4). The final multivariate model demonstrated significant negative correlation with VAS pain score average (β: − 0.094, 95% CI − 0.175 to − 0.013; p = 0.024), patients with higher VAS pain scores have decreased facilitation. Additionally, we found a positive correlation between facilitation and the WOMAC pain subscale score (β: 0.062, 95% CI 0.011 to 0.113, p = 0.018), facilitation is increased the more pain patients report through this scale in contrast with the VAS correlation. Finally, the HADS anxiety subscale was negatively correlated with ICF (β: − 0.039, 95% CI − 0.078 to − 0.0007, p = 0.046), meaning higher the anxiety level smaller the ICF. Additional variables were included into the model as they were significant confounders such as gender, BMI, and pain threshold (Table 4).

**Table 4:** Univariate analysis of clinical and sociodemographic variables associated with ICF and final model of multivariate analysis with significant associated variables with ICF.

Variables	β-coefficient	95% CI	Unadjusted p-value	
Age	− 0.0047	− 0.016 to 0.007	0.422	
Gender	− 0.24	− 0.53 to 0.052	0.106	
Time of ongoing pain (months)	0.00105	− 0.00005 to 0.002	0.062	
Weight (kg)	− 0.00067	− 0.008 to 0.006	0.852	
Height (m)	0.656	− 0.52 to 1.83	0.272	
BMI	− 0.00866	− 0.029 to 0.012	0.412	
**Kellgren–Lawrence classification**				
Right	− 0.044	− 0.139 to 0.051	0.357	
Left	− 0.036	− 0.13 to 0.060	0.454	
Average total	− 0.046	− 0.147 to 0.055	0.364	
**Knee arthroplasty (no)**				
Right	− 0.32	− 1.14 to 0.49	0.431	
Left	0.12	− 0.55 to 0.79	0.722	
Ten-minute walking test	− 0.00738	− 0.023 to 0.009	0.366	
Six-minute walking test (distance in meters)	0.00080	− 0.0002 to 0.002	0.12	
Timed up-and-go	− 0.0059	− 0.020 to 0.008	0.421	
Berg balance scale	0.0053	− 0.008 to 0.019	0.437	
Epworth sleepiness scale	− 0.0138	− 0.033 to 0.005	0.146	
Medical research council scale bilateral average	0.097	− 0.086 to 0.28	0.296	
WOMAC stiffness	− 0.022	− 0.078 to 0.033	0.429	
WOMAC physical function	− 0.0044	− 0.012 to 0.003	0.281	
WOMAC pain	− 0.010	− 0.038 to 0.018	0.468	
**Pain pressure threshold (kPa)**				
Knee				
Right	0.026	− 0.017 to 0.069	0.240	
Left	0.026	− 0.019 to 0.070	0.255	
Bilateral	0.026	− 0.018 to 0.071	0.239	
Upper limb				
Right	− 0.007	− 0.06 to 0.047	0.807	
Left	− 0.0117	− 0.006 to 0.04	0.656	
Bilateral	− 0.010	− 0.065 to 0.045	0.72	
Conditioned pain modulation (kPa)	− 0.0559	− 0.159 to 0.048	0.287	
**Visual analogue scale**				
Right	− 0.042	− 0.08 to − 0.0041	0.03	
Left	− 0.021	− 0.061 to 0.018	0.285	
Bilateral	− 0.0598	− 0.11 to − 0.0072	0.026	
Pain catastrophizing scale	− 0.002	− 0.0126 to 0.00787	0.645	
HAM-D scale	− 0.014	− 0.034 to 0.0054	0.152	
**Hospital Anxiety and depression scale**				
Anxiety	− 0.027	− 0.0526 to − 0.0007	0.044	
Depression	− 0.012	− 0.043 to 0.019	0.458	
**Montreal cognitive assessment**				
Education level (years)	0.009	− 0.021 to 0.04	0.541	
Total score	0.007	− 0.015 to 0.30	0.53	

*Model 3.* multivariate analysis for ICF			Adjusted p-value	R-Squared: 0.1505
Gender	− 0.167	− 0.52 to 0.184	0.346	
BMI	− 0.010	− 0.037 to 0.016	0.423	
CPM	− 0.090	− 0.199 to − 0.019	0.105	
WOMAC Pain	0.062	0.011 to 0.113	0.018	
VAS Bilateral	− 0.094	− 0.175 to − 0.013	0.024	
HAD-Anxiety	− 0.039	− 0.078 to − 0.0007	0.046	

*BMI* Body Mass Index, *WOMAC Pain/Stiffness/Function* Western Ontario and MacMaster Universities Osteoarthritis Index Pain/Stiffness/Function section, *HAM-D* Hamilton Depression Rating Scale, *HAD-Anxiety* Hospital Anxiety and Depression Scale—Anxiety section.

### Motor threshold (MT)

In the final, multivariate model, significance was conveyed in age (p = 0.037), the WOMAC pain score (p = 0.035), the bilateral average of the VAS pain score (p = 0.013), and the anxiety section of the Hospital Anxiety and Depression Scale (HADS) (p = 0.034) (Table 5). A linear, inverse relationship was found between age and motor threshold (β: − 0.306, 95% CI − 0.59 to − 0.018, p = 0.037) meaning, there is a decline in motor threshold as one gets older. An inverse relationship was also seen with the HADS for anxiety (β: − 0.813, 95% CI − 1.56 to − 0.062, p = 0.034); the higher the patient's anxiety, according to their score, the lower their motor threshold. Moreover, in the scope of pain, different significant relationships were identified in consonance with the assessment performed; the more pain a patient feels, as reported by their WOMAC score, the higher their motor threshold conveying a positive correlation between the assessment and the marker (β: 1.029, 95% CI 0.075 to 1.980, p = 0.035). Notwithstanding, the higher pain reported by patients on their VAS score, the lower their motor threshold, depicting a negative correlation (β: − 2.003, 95% CI − 3.566 to − 0.438, p = 0.013). Thus, a paradoxical difference between the two pain-rating scales can be observed. We identified the following confounders: gender, conditioned pain modulation (CPM), and time of ongoing pain, which were included into the model.

**Table 5:** Univariate analysis of clinical and sociodemographic variables associated with MT and final model of multivariate analysis with significant associated variables with MT.

Variables	β-coefficient	95% CI	Unadjusted p-value	
Age	− 0.15	− 0.38 to 0.073	0.182	
Gender	− 2.701	− 8.57 to 3.17	0.364	
Time of ongoing pain (months)	− 0.009	− 0.031 to 0.013	0.405	
Weight (kg)	0.17	0.036 to 0.31	0.014	
Height (m)	32.866	10.16 to 55.57	0.005	
BMI	0.19	− 0.22 to 0.60	0.362	
**Kellgren–Lawrence classification**				
Right	− 1.20	− 3.09668 to 0.69	0.21	
Left	− 0.938	− 2.87 to 0.996	0.338	
Average total	− 1.18	− 3.21 to 0.848	0.251	
**Knee arthroplasty (no)**				
Right	7.22	− 9.066 to 23.518	0.381	
Left	− 7.11	− 20.456 to 6.23	0.293	
Ten-minute walking test	− 0.209	− 0.526 to 0.108	0.194	
Six-minute walking test (distance in meters)	0.019	− 0.0006 to 0.039	0.057	
Timed up-and-go	− 0.26	− 0.548 to 0.018	0.067	
Berg balance scale	0.176	− 0.0897 to 0.44	0.192	
Epworth sleepiness scale	0.101	− 0.275 to 0.478	0.595	
Medical research council scale bilateral average	4.827	1.28 to 8.37	0.008	
WOMAC stiffness	− 0.648	− 1.75 to 0.45	0.247	
WOMAC physical function	− 0.0536	− 0.21 to 0.107	0.509	
WOMAC pain	− 0.17	− 0.53 to 1.60	0.384	
**Pain pressure threshold (kPa)**				
Knee				
Right	0.963	0.117 to 1.809	0.026	
Left	0.795	− 0.087 to 1.678	0.077	
Bilateral	0.912	− 0.034 to 1.791	0.042	
Upper Limb				
Right	0.53	− 0.916 to 0.48	0.324	
Left	0.12	− 0.747 to 1.437	0.815	
Bilateral	0.34	− 0.27 to 0.478	0.532	
Conditioned pain modulation (kPa)	− 1.50	− 3.489 to 0.48	0.135	
**Visual analogue scale**				
Right	− 0.71	− 1.479 to 0.057	0.069	
Left	− 0.246	− 1.0347 to 0.54	0.537	
Bilateral	− 0.898	− 1.957 to 0.16	0.095	
Pain catastrophizing scale	− 0.94	− 0.298 to 0.109	0.361	
HAM-D scale	0.24	− 0.158 to 0.64	0.234	
**Hospital anxiety and depression scale**				
Anxiety	− 0.24	− 0.77 to 0.28	0.358	
Depression	0.0219	− 0.065 to 0.649	0.945	
**Montreal cognitive assessment**				
Education Level (years)	0.178	− 0.43 to 0.79	0.564	
Total score	0.13	− 0.32 to 0.586	0.569	

*Model 4.* Multivariate Analysis for MT (µV)			Adjusted p-value	R-Squared: 0.1994
Age	− 0.306	− 0.590 to − 0.018	0.037	
Gender	− 1.430	− 7.920 to 5.060	0.661	
Time of ongoing pain (months)	− 0.009	− 0.034 to 0.0158	0.463	
Conditioned pain modulation	− 1.510	− 3.587 to 0.560	0.150	
WOMAC pain	1.029	0.075 to 1.980	0.035	
Visual analogue scale average	− 2.003	− 3.566 to − 0.438	0.013	
Hospital anxiety scale	− 0.813	− 1.560 to − 0.062	0.034	

*BMI* Body Mass Index, *WOMAC Pain/Stiffness/Function* Western Ontario and MacMaster Universities Osteoarthritis Index Pain/Stiffness/Function section, *HAM-D* Hamilton Depression Rating Scale, *HAD-Anxiety* Hospital Anxiety and Depression Scale—Anxiety section.

### Motor evoked potential (MEP)

Motor evoked potential (MEP) univariate analysis only shows a positive significant correlation between left total knee arthroplasty (β: 2.664, p = 0.001, 95% CI: 1.097 to 4.230) and MEP in OA (Table 6). Nonetheless, this significance is not upheld in the final multivariate model. Moreover, no other significant findings are portrayed in the final MEP multivariate model, indicating no cognitive-emotional, functional, or pain relationship between the chronic pain related to OA and MEP.

**Table 6:** Univariate analysis of clinical and sociodemographic variables associated with MEP.

Variables	β-coefficient	95% CI	Unadjusted p-value
Age	− 0.0016	− 0.030 to 0.027	0.910
Gender	− 0.354	− 1.076 to 0.368	0.334
Time of ongoing pain (months)	− 0.0008	− 0.003 to 0.002	0.585
Weight (kg)	− 0.004	− 0.021 to 0.014	0.668
Height (m)	0.0657	− 2.240 to 3.555	0.654
BMI	− 0.021	− 0.072 to 0.029	0.406
**Kellgren-Lawrence classification**			
Right	− 0.011	− 0.246 to 0.223	0.922
Left	− 0.086	− 0.0284 to 0.111	0.386
Average total	− 0.073	− 0.281 to 0.134	0.486
**Knee arthroplasty**			
Right	0.194	− 1.817 to 2.205	0.849
Left	2.664	1.097 to 4.230	0.001
Ten-minute walking test	0.001	− 0.038 to 0.040	0.951
Six-minute walking test (distance in meters)	− 0.0004	− 0.003 to 0.002	0.717
Timed up-and-go	− 0.001	− 0.037 to 0.034	0.936
Berg balance scale	0.001	− 0.032 to 0.034	0.953
Epworth sleepiness scale	0.006	− 0040 to 0.052	0.795
Medical research council scale bilateral average	0.210	− 0.239 to 0.659	0.356
WOMAC stiffness	− 0.055	− 0.192 to 0.081	0.425
WOMAC physical function	− 0.006	− 0.026 to 0.136	0.532
WOMAC pain	− 0.012	− 0.082 to 0.056	0.719
**Pain pressure threshold (kPa)**			
Knee			
Right	− 0.037	− 0.142 to 0.068	0.490
Left	− 0.001	− 0.110 to 0.108	0.981
Bilateral	− 0.020	− 0.129 to 0.089	0.713
Upper Limb			
Right	0.054	− 0.145 to 0.119	0.846
Left	0.054	− 0.084 to 0.172	0.494
Bilateral	0.058	− 0.111 to 0.171	0.675
Conditioned pain modulation (kPa)	0.087	− 0.167 to 0.341	0.499
**Visual analogue scale**			
Right	0.010	− 0.086 to 0.105	0.841
Left	− 0.015	− 0.112 to 0.082	0.763
Bilateral	− 0.004	− 0.136 to 0.127	0.946
Pain catastrophizing scale	0.0004	− 0.025 to 0.026	0.973
HAM-D scale	− 0.009	− 0.058 to 0.041	0.724
**Hospital Anxiety and depression scale**			
Anxiety	− 0.006	− 0.071 to 0.058	0.844
Depression	− 0.001	− 0.078 to 0.076	0.971
**Montreal cognitive assessment**			
Education level (years)	− 0.060	− 0.134 to 0.014	0.111
Total score	− 0.010	− 0.067 to 0.045	0.699

*BMI* Body Mass Index, *WOMAC Pain/Stiffness/Function* Western Ontario and MacMaster Universities Osteoarthritis Index Pain/Stiffness/Function section, *HAM-D* Hamilton Depression Rating Scale, *HAD-Anxiety* Hospital Anxiety and Depression Scale—Anxiety section.

## Discussion

### Summary of the study’s main findings

This study aimed to explore the association of different sociodemographic and clinical variables and the cortex excitability conveyed by MT, MEP, CSP, SICI, and ICF TMS markers in subjects with chronic pain due to OA. This is one of the first studies exploring the association between TMS measurements of cortex excitability and clinical and demographic variables in a large sample of chronic knee OA pain^18^, looking to understand better how maladaptive mechanisms linked with chronic pain work and may be expressed by these neurophysiological markers. Our main findings showed important relationships between clinical and demographic variables and cortical excitability markers indexed by TMS. Multivariate analyses conveyed age, OA severity, anxiety, cognitive function, and pain measured by the WOMAC scale as significant variables associated with cortical inhibition (indexed by SICI and CSP), and anxiety and pain associated with intracortical facilitation (represented by ICF and MT) in chronic knee OA pain patients.

### Intracortical inhibition and age

We found a direct relationship between age and intracortical inhibition, showing that as subjects with chronic pain get older, inhibition decreases. As discussed above, these findings are similar with our other models, relating the disinhibited state with older patients. Intracortical inhibition develops gradually during the first two decades of life, is maximal during young adulthood, and declines with age^19,20^. In these studies, Mall et al. have shown that children presented a higher ICI ratio (0.71) when compared with adolescents (0.33), and adults (0.21), meaning that the cortical inhibition tend to decrease with age. Moreover, Peinemann et al. main findings have supported and demonstrated a linear age-related decline in intracortical inhibition in healthy subjects (r = 0.234). It is difficult here to detangle whether this relationship between age and ICI is also enhanced by their OA diagnosis. However, these results show that the mechanism of ICI decreases over time and thus may contribute to worse adaptation in older age.

Additionally, the cortical silent period showed an inverse association with age, this means that as subjects get older, the duration of this period decreases, thus there is less intracortical inhibition. These results are therefore similar to those found regarding the SICI stated above and with some other studies that elderly patients present less cortical inhibition^21^. An important question that we may answer is that this relationship is not related to the time of ongoing pain since this variable was not significant in our two models (SICI and CSP).

However, age was not seen associated with ICF and MEP in the present study. This lack of association was present in previous research^20,22^. Therefore, the relationship between the age and intracortical excitability seems to be specific for intracortical inhibition. In fact, results seem to be mixed with some studies showing no association, some of them show less ICF in older people^23^, and, in others older subjects have shown a higher short ICF^24^. Thus, more powerful analyses may help to understand this relationship better. Likewise, our study did not detect a relationship between age and MEP. Given that MEP is a general measure of corticospinal excitability and thus has a low specificity, this result also favors the notion that an age-related decrease in intracortical inhibition is specific for this population.

On the other hand, previous studies have shown that the aging process is correlated with the decreasing in the MEP in health subjects^25^. One limitation of this study is the uneven age distribution, since OA is a disease present in a specific age range^26^. Therefore, a study with a more uniform distribution of age in a chronic pain population is needed to explore more consistently the relationship of MEP and age in this population.

### Intracortical excitability, VAS and WOMAC Pain

Regarding pain, one of the main findings of our study is that there was a direct association with the WOMAC scale for pain, showing that pain-related activity is associated with lower cortical inhibition. These results corroborate with our hypothesis that patients with less physical activity (in this case because of pain) have less cortical inhibition and thus likely less cortical compensation that may lead to more pain^27^. Our findings support the idea that higher cortical excitability is present in mechanisms of knee osteoarthritis. The relationship between this potential disinhibited state and chronic pain was found in previous studies in patients with chronic pain due to other etiologies, like fibromyalgia^18,28^.

However, VAS was not associated with measures of intracortical inhibition (CSP and SICI). This is an interesting finding, besides expected. We have shown in several studies that pain intensity does not correlate with ICI^6,9^. In a review, Santos et al. analyzed ICI and CSP in studies targeting individuals with limb amputation and most of these showed no relationship between cortical excitability and pain intensity^9^. Additionally, Teixeira et al. were not able to find any association between changing in cortical excitability and pain intensity, analyzing longitudinal data from a clinical trial targeting subjects with phantom limb pain^6^. However, intracortical inhibition seems to be a marker of adaptation to chronic pain^18,29^. It may indicate a dysfunctional sensory system and central adaptation. Although, after this system is overcome then pain intensity is likely modulated by other factors, such as emotional affective factors that are likely associated with VAS as shown by other studies^30,31^.

Furthermore, our study did not find any significant relationship between the cortical silent period and the pain scales used to evaluate knee OA pain in this study, including WOMAC pain. Although we expected that CSP and SICI would have similar results as both measure intracortical inhibition, they measure different inhibitory pathways, while SICI measures GABA-A pathways, CSP is related to the GABA-B system^32^, what could explain the divergent results. Other studies also relate inverse correlation between CSP and pain modulatory systems, relating the relationship with central sensitization and the existing inflammatory process of knee OA^21^.

Finally, in our ICF model, similar to the findings for the MT measurement, a negative correlation is found with VAS pain scores and ICF, juxtaposing the WOMAC findings. Given that both VAS and WOMAC are validated and sensitive scales to evaluate pain in knee OA^33^, their conflicting correlations found in pain according to MT and ICF assessments suggest that these two assessments evaluate different aspects of pain. The WOMAC scale consists of five items, all of which ask the subject about pain while performing a specific movement or motor function^34^. On the other hand, VAS is a single-question assessment that inquiries about pain in a broadened sense, which relates deeply to the subject’s perception of pain as a whole, rather than when performing specific tasks^30^. WOMAC pain is a subscale that it is not usually use in isolation for clinical purposes. Although, both scales assess pain including all biopsychosocial domains. VAS and WOMAC pain scales are weighting differently some domains. WOMAC gives more weight to the sensorimotor and movement-related domains of pain perception (although the other domains are still in play). On the other hand, VAS asks a single question about the pain intensity, which encompasses all the biopsychosocial aspect of pain. The different contexts of evaluation brought by these two pain scales could explain the contradicting results in our model. Additionally, our finding regarding the VAS reinforces previous studies’ results, in which the same negative association between the ICF and VAS in patients with phantom limb pain was found^6,35,36^. All these finds may show that the VAS is not a good tool regarding cortex excitability.

### Cartilage damage (Kellgren Lawrence classification) and intracortical inhibition

We found an interesting association between SICI and Kellgren Lawrence Classification, this means that higher K-L scores are associated with more inhibition. The K–L score indicates the amount of cartilage damage. This correlation therefore shows that intracortical inhibition appears to be a mechanism of compensation to the amount of cartilage damage. This also provides a potential explanation for why K-L is not the main determinant of pain. In other words, some subjects with low pain and high K-L have likely larger cortical inhibition. This could be a body temptation to reduce the perception of pain in those patients with the more severe disease. Moreover, this result corroborates with what was found in the CSP model, as stated below.

A significant positive correlation has been found between the Kellgren-Lawrence Classification for OA severity and the cortical silent period meaning that the higher severity of OA, the longer the cortical silent period, meaning more intracortical inhibition. OA severity has been seen to respond differently to chronic pain mechanisms including the process of central pain sensitization^37^. This is the main reason why central sensitization is still not a broadly recognized mechanism of knee OA pain. Additionally, studies have reported that patients with low pain sensitivity and advanced disease do not respond as well to brain stimulation techniques, indicating that central sensitization might not be a mechanism for all individuals with knee osteoarthritis^37^.

On the other hand, in our study, no relationship between the OA severity (indexed by K-L classification) and ICF, MT, or MEP was found. This result again shows that intracortical inhibition seems to be a specific marker of compensation to OA, rather than a general change in excitability.

### Emotional affective system and intracortical excitability

Anxiety has been intimately related to chronic pain and the modulation of noxious descending pathways. Our results convey a significant negative relationship between anxiety, SICI and ICF. This goes against other studies that convey increased cortical facilitation and decreased inhibition in individuals with psychiatric disorders, conveying dysfunctional GABAergic pathways and increased glutamatergic NMDA activity^38^. An explanation for our conflicting findings may be that the literature conveys dysfunctional pathways in subjects with diagnosed psychiatric disorders^38^. Meanwhile, our sample only conveys subjects with mild anxiety traits (as evidenced by the mean HAD-Anxiety scale core of 5.92). Thus, the anxiety levels conveyed by our sample are not enough to trigger malfunctioning cortical pathways and instead, might offer a compensatory mechanism, suggesting a bimodal model of anxiety related to cortical inhibition in pain settings. This model is seen in a study done in healthy subjects depicting that subjects with different levels of anxiety convey different tolerances to pain; patients sensible to anxiety traits have higher pain tolerance and higher levels of anxiety are related to hyperalgesia^39^.

Although, given we were assessing the primary motor cortex, we found a relatively minor influence of anxiety and depression in intracortical excitability in ICI and ICF, and no association with CSP, MT, and MEP. These results are similar to previous studies that tested the association of emotional measurement and motor cortical excitability^40,41^. This shows therefore that the effects of the emotional system on pain are independently on the compensation effects of the primary motor cortex on pain.

### Cognition and intracortical excitability

Several studies have contributed to understanding the mechanisms behind the relationship between cognition and cortical excitability in the chronic pain field^42^. The literature also contributes to the understanding of the relationship between cognition and cortical inhibition. We found that MoCA is negatively correlated with CSP, meaning lower MoCA scores indicate longer CSPs. This finding correlates to other results that link cognitive impairment with longer duration CSP found in patients with mild cognitive impairment and Alzheimer's disease^43^. It is thought that CSP directly displays GABA-B slow inhibition activity which is activated by glutamatergic receptors^44^. In individuals with cognitive impairment, fast glutamatergic transmissions stimulate GABA-b slow-mediated inhibition which prolongs CSP, explaining the negative correlation between MoCA Scores and CSP^43,45^. We also hypothesize that the relationship between shorter CSP (therefore, less inhibition) and higher MoCA scores may be related to more avoidance of painful movements in individuals with higher cognition. Subjects with higher cognitive function might be more aware of movements that lead to pain and thus avoid moving at all. The lack of movement “atrophies” cortical inhibition, making individuals with higher cognition feel more pain.

### Motor threshold (MT)

The motor threshold presented an inverse association with age, indicating that older the age, lower the MT. One potential interpretation is that as subjects get older and loose capacity of cortical inhibition, they therefore have a lower motor threshold due to inhibitory deficits. These findings reinforce what was found in previous studies in healthy subjects^46–48^ and what we found in our following models using SICI and CSP. In the context of pain, our results convey contrary direction relationships between MT vs. VAS and vs. WOMAC. An inverse relationship is seen between MT and pain reported through the VAS scale, meaning that the more pain reported by a patient on this scale, the lower their motor threshold; thus, confirming the notion of a lower adaptation and thus more pain. This finding relates to other studies in which negative correlations have been found between resting MT and higher pain levels^49^. Finally for WOMAC the relationship is positive meaning that more pain on this instrument resulted in higher MT. This may also indicate adaptation of subjects trying to move and thus likely adjusting the motor threshold. Noteworthy, we also need to underscore it is known that MT has a high variability inter-subjects^50^, thus results from this variable need to be interpreted with this caveat.

### Quantitative sensory testing (QST) and intracortical excitability

Our models were not able to find a relationship between intracortical excitability and quantitative sensory testing measurements (CPM and pain thresholds). This is one of the most powered studies to analyze this relationship in a multivariate model adjusting for clinical factors that influence both markers, such as age^20,51^, gender^52^, emotion^53^, and cognitive variables^54^. However, the relationship between the CPM and cortical excitability is not well understood. Some studies have found a relationship between these markers on chronic pain populations^55^. We hypothesize that the CPM is associated with excitability and inhibition of central pain processing system through multiple independent pathways; therefore, we do not expect always a high correlation with motor cortex excitability in chronic pain.

### Limitations

Considering its exploratory nature, our study has some limitations. The lack of a control group in our study prevents us from assuming that the associations found between the cortical excitability measures and sociodemographic and clinical variables indeed pertain to individuals with OA chronic pain. However, since we wanted to understand the factors in OA that drives intracortical excitability (especially inhibitory markers), adding a control group would limit the scope of our associated factors (we could not include disease severity or pain intensity in our statistical models). Moreover, we did not perform corrections for multiple analyses, which could have led to an increase in type two error in our results. Nonetheless, given this is an exploratory study, significant results found in the multivariate analyses still remain valid and compelling for further exploration.

## Conclusion

In conclusion, our study could identify clear associations of clinical and demographic variables and cortical excitability in patients with chronic pain caused by knee OA. These associations showed the fundamental role of intracortical inhibition as a marker of adaptation to chronic pain such as that patients with higher intracortical inhibition (likely subjects with more compensation) are younger, have greater cartilage degeneration (indexed by Kellgren-Lawrence severity classification), and have less pain in the WOMAC scale. Also, it is important to note that this variable is not significantly affected by emotional factors and pain measured by VAS. Other markers as MT and CSP corroborate with the primary finding. However, our study has some limitations regarding our methodology and the generalizability of our results. The information revealed here helps us to understand the role of cortex excitability and its changes and characteristics as a biomarker in the pain field. Finally, more research is needed with broader and more general samples to bring more consistency for the role of these biomarkers.

## Methods

### Subjects

Data were collected from 107 patients with knee osteoarthritis (OA). These patients were obtained from an ongoing, prospective cohort study titled “Deficit of Inhibition as a Marker of Neuroplasticity (DEFINE study) in rehabilitation”^56^. The DEFINE protocol and this study were approved by the Research and Ethical Comitee of Hospital das Clínicas da Faculdade de Medicina da Universidade de São Paulo (HC FMUSP) **(**Registration number: 86832518.7.0000.0068). All the proceedings and methods of this study are in accordance with Brazilian research ethics regulations and the Declaration of Helsinki. We included male and female participants, 18 years of age and older that fulfill eligibility criteria for the conventional rehabilitation program of the Instituto de Medicina Fisica e Reabilitacao (IMREA). The diagnosis of osteoarthritis was made through a clinical and radiological (magnetic resonance imaging or computerized tomography; or bilateral knee radiography) diagnosis of knee OA. Individuals with bilateral symptoms who had undergone total knee replacement surgery were included in our sample as they still had unilateral chronic pain related to osteoarthritis (on the other knee) and made up less than 5% (six patients, VAS Pain mean = 4.78, SD = 0.77) of our sample. Individuals with bilateral total knee replacement surgery were excluded. Subjects were excluded if they were pregnant, have active OA with clinical manifestations in joints other than the knee, or if they had any other clinical or social conditions that interfere with the patient’s participation in the rehabilitation program. Characteristics of included knee OA patients are summarized in Table 1.

### Study design

This was a cross-sectional study made up of patients with the diagnosis of knee OA from the DEFINE longitudinal cohort study^56^. Patients admitted to the IMREA’s conventional rehabilitation program with knee OA were invited to participate in the study and included after signing the informed consent form. Patients who agreed to participate in the study underwent a series of neurophysiological and functional assessments before the IMREA rehabilitation program. This program is characterized by an individualized approach, considering the injury’s etiology, the type of disabilities the patient has, general clinical conditions, likely prognosis, and the patient’s socioeconomic factors.

### Clinical and functional assessments

Information regarding the participants’ age, gender, time of ongoing pain, height, weight, body mass index, education and cognitive levels were collected for analysis in our regression models with several instruments that allow the global assessment of participants’ pain, motor, cognitive, or emotional function. A summary of all assessments can be seen in the supplementary information file. Some scales, such as cognitive, sleep, and mood scales, were used to characterize the study’s sample, as well as for the management of confounding variables on the multivariate statistical model. Assessments were preferably carried out by the same evaluator. Evaluators were trained to standardized questionnaire applications to reduce assessor bias and variability.

### Neurophysiological assessment

The Magstim Rapid® stimulator (The Magstim Company Limited, UK) and a 70 mm coil in figure-of-eight were used, we positioned tangentially to the skull and at an angle of 45 degrees about the sagittal line. The muscular response to the stimulus applied to the motor cortex was recorded using surface electromyography (EMG) with Ag/AgCl electrodes positioned on the target muscle (first dorsal interosseous (FDI) muscle of the hand) and the grounding electrode positioned on the wrist.

Bilateral upper limb assessment was performed. The motor area corresponding to the FDI is the most used motor cortical area in cortical excitability studies due to its greater accuracy^57^. To locate the cortical area of the hand, it was initially identified from the vertex (intersection between the nasion-inion lines and zygomatic arches). Then, for the identification of the probable hot spot, a mark was made 5 cm from the vertex towards the ear tragus in the coronal plane. The hotspot was determined as the location with the highest and most stable MEP amplitudes over the FDI.

The rMT was defined as the minimum intensity necessary for a single TMS pulse on the hot spot to generate an MEP, with at least 50 μV peak to peak amplitude, in 50% of attempts^58^. RMT was used as an indirect measure of cortical excitability. In addition, the following measures were used: MEP, in which ten MEP were recorded, with an interval of approximately 7 s between stimuli; cortical silent period (CSP) was measured, which represents the temporary suppression of electromyographic activity during a sustained motor evoked potential voluntary contraction. Finally, we performed paired-pulse protocols of intracortical inhibition (SICI), which was assessed by interstimulus intervals of 2 ms; and intracortical facilitation (ICF) assessed by 10 ms interim stimulus intervals^58^. For more details on the neurophysiological assessment please see the study protocol published elsewhere^56^.

For the measurement of neurophysiological markers through TMS, we combined the rMT, CSP, SICI, ICF, and MEP results to obtain a bi-hemispheric average. This approach can be justified due to the bi-hemispheric nature of pain perception^59^; besides, most of our sample includes patients with bilateral knee OA. We then analyzed the relationship between the bi-hemispheric average of these neuro markers with possible associated variables to their behavior (markers magnitude and direction), including clinical and sociodemographic subject characteristics.

### Statistical analysis

Descriptive statistics were used to characterize the study sample, categorical variables were summarized by frequency and percentiles and continuous variables were analyzed by mean and standard deviation (SD). Some of the scales, such as those for mood, pain, cognition, and sleep, were used to characterize the sample, in addition to possibly being used to control confounders in the multivariate model.

We aimed to model the relationship between sociodemographic and clinical variables, as independent variables, and the cortical excitability variables of MT, SICI, ICF, SP, and MEP recorded by TMS, as dependent variables. To select the model covariates, we performed univariate regression analysis for each variable in which variables that reached a p-value < 0.25 were added in the multivariate model. Univariate models were used to not only better select the most significant variables to the final model, but also to minimize the number of tests required to achieve the final model^60^. Then, to investigate the association between cortex excitability and clinical variables, five separate multivariate linear regression models were created using MT, SICI, ICF, SP, and MEP, as dependent variables, and the significant clinical variables from the univariate analysis. We used a “purposeful selection” method in the process of variable selection to build the models, variables were removed from the models if they were not significant, not a confounder, or not clinical important, thus, we could include in the final models, variables that were not significant in the univariate analysis^61^. Variables were considered as confounders if they changed the β coefficient of the dependent variable of more than 10%, compared with the previous model. Lastly, to determine the best fit model, the Akaike’s information criteria was used. Models displayed in the results section are the final multivariate models found through this method.

During the modeling process we test linearity assumption visually comparing the scatterplot of each independent variable and a superimposed regression line plot. The homoscedasticity assumption was checked by visual inspection of the scatterplot of the standardized fitted values and standardized residuals^62^. We defined outliers as values greater than 3 SDs away from the average scores and performed a sensitivity analysis if detected. Finally, we tested the residuals normality using histograms and the Shapiro–Wilk test^63^ and assumed normality based on the central limit theorem for those with approximate normal distribution. Because this was an exploratory study and to minimize the risk of type II errors, no correction for multiple comparisons was done. We used Stata Statistical Software 15 (Stata Corp LLC) for the statistical analyses.

## Supplementary Information

Supplementary Information.
