# Supplementary material for: Increased motor cortex inhibition as a marker of compensation to chronic pain in knee osteoarthritis
Source: Sci Rep. 2021 Dec 14;11:24011. doi: 10.1038/s41598-021-03281-0 (PMC8671542; doi:10.1038/s41598-021-03281-0)
Supplement: Supplementary file 1 — Supplementary Information. [file 41598_2021_3281_MOESM1_ESM.docx]

**Supplementary information**

**Functional Clinical Assessment Description and Application**

*Adapted from our protocol under review – Deficit of inhibition as a marker of neuroplasticity - DEFINE study - in rehabilitation: a longitudinal cohort study protocol - Sims et al. 2021^1^

Montreal Cognitive Assessment (MOCA): Screens the patient’s dominance over their cognitive function’s executive function, visuo-spatial ability, memory, attention, concentration, occupational memory, language, and temporal and spatial orientation. The scale has a maximum score of 30 points and an application time of approximately 10 minutes.^2^

Conditioned pain modulation (CPM): Through intense heterotopic stimulation, this test evaluates a patient’s endogenous modulation response to pain. Subjects will immerse one of their hands into a recipient containing cold water (10-12oC) for one minute. After 30 seconds of immersion, the Visual Analogue Scale (VAS) will be presented to patients so that they can indicate their pain level, referring to the submerged hand. Subsequently, three algometric measures will be taken (spaced between 15 seconds) for the contralateral hand. After an interval of approximately 10 minutes (time for hand to return to normal body temperature), the other hand will be immersed in the recipient, and follow the previously stated protocol.^3^

Pressure Pain Threshold (PPT): This test uses an algometer to define the minimum amount of pressure that triggers pain in pre-established regions (thenar region, medium deltoid, and region located one inch above the patella).^4^

Pain Catastrophizing Scale: Made up of nine items staggered on a Likert scale, varying from 0-5 points related to the words “almost never” and “almost always” on the extremities. The total score is the sum of the items divided by the number of answered items, with the minimum achievable score being 0 and the maximum 5. Higher scores indicate the presence of catastrophizing thoughts.^5,6^

Visual Analogue Scale (VAS) for Pain: Made up of a 10 cm straight line on a piece of paper. Written on one of its ends is the phrase “no pain” and on the other “maximum pain”. Each subject will be asked to mark their discomfort level on the VAS line. The closer the mark is to the scale’s origin (zero centimeters), the lower the patient’s pain level. On the other hand, the closer the mark is to the scale’s end (ten centimeters), the higher the patient’s pain level. Instructions for the patient will be “Identify the amount of pain experienced in the last 48h and make a mark perpendicular to the ‘no pain’ – ‘maximum pain’ line”.^7^

6-minute and 10 meters gait test:^8^

10-meter gait test: aims to assess a patient’s short-distance walk speed. It is

recommended that the subject walks 14 meters so that the 2 initial and final meters be disregarded. The subject will walk at their normal speed.

6-minute gait test: evaluates the maximum distance a subject can walk on a plane, rigid surface in six minutes through a 30-meter track. It is recommended that this be a 30-meter walk, lapped every 3 meters in which turning points are set with a cone.

Timed Up and Go (TUG): This test evaluates an individual’s mobility level, measuring the amount of time it takes for the subject to stand up from a chair without using their arms, walk a 3-meter distance, turn 180o and get back to sit on the chair.^9^

Medical Research Council Scale (MRC): It is an assessment performed quickly that classifies muscle strength in 5 different degrees. For the upper limb, shoulder abduction, elbow flexion and extension, and wrist and finger extension will be evaluated. As for the lower limb, knee extension and flexion will be evaluated.^10^

Berg Balance Scale: this scale is made up of 14 tasks that assess static and dynamic balance, through tasks such as reaching, turning, transferring, standing, and standing up. The score for each item ranges from 0-4, determined by the ability to perform the task, and the maximum score is 56 points.^11^

Epworth sleepiness scale: Evaluates the degree of daytime sleepiness. It is a self-applied questionnaire that evaluates the probability of falling asleep in 8 everyday situations.^12^

Hamilton Depression Rating Scale (HAM-D): The instrument investigates how the patient has been feeling in the last seven days, including the day of application; it consists of 17 items, which can be scored on a Likert scale ranging from 0 to 2 or 0 to 4, depending on the intensity of the symptom. The total number of points varies between 0 and 52 points. To verify the presence of depression, the scores must add up to at least 8 points in the original version.^13^

Hospital Anxiety and Depression Scale (HADS): A 14-item scale that quantifies and qualifies symptoms of anxiety and depression. The scale contains 14 multiple-choice questions. It consists of two subscales, one for anxiety and another for depression, with seven items each. The global score in each subscale ranges from 0 to 21. It is intended to detect mild degrees of affective disorders in non-psychiatric environments. The patient is asked to respond based on how he felt during the last week.^14^

Western Ontario and McMaster Universities Osteoarthritis Index (WOMAC): Scale used to measure pain, stiffness, and function in patients with knee and hip osteoarthritis. The questionnaire consists of 24 items, divided into 3 subscales: pain, addressing 5 items, stiffness, 2 items, and function, 17 items.^15^

Kellgren-Lawrence Radiographic Classification of OA: it is a method of classifying the severity of knee osteoarthritis (OA), which uses five degrees: 0 (without osteoarthritis) to 4 (large osteophyte, marked narrowing of the joint space, severe sclerosis, and definite deformity of bony extremities).^16^

**REFERENCES**

1 Simis, M. *et al.* Deficit of inhibition as a marker of neuroplasticity (DEFINE study) in rehabilitation: a longitudinal cohort study protocol. *Frontiers in Neurology* **12**, 1193 (2021).

2 Freitas, S., Simões, M. R., Alves, L. & Santana, I. Montreal Cognitive Assessment (MoCA): normative study for the Portuguese population. *J Clin Exp Neuropsychol* **33**, 989-996, doi:10.1080/13803395.2011.589374 (2011).

3 Streff, A., Michaux, G. & Anton, F. Internal validity of inter-digital web pinching as a model for perceptual diffuse noxious inhibitory controls-induced hypoalgesia in healthy humans. *Eur J Pain* **15**, 45-52, doi:10.1016/j.ejpain.2010.05.011 (2011).

4 Reidler, J. S. *et al.* Effects of motor cortex modulation and descending inhibitory systems on pain thresholds in healthy subjects. *J Pain* **13**, 450-458, doi:10.1016/j.jpain.2012.01.005 (2012).

5 Sullivan, M. J. L., Bishop, S. R. & Pivik, J. The Pain Catastrophizing Scale: Development and validation. *Psychological Assessment* **7**, 524-532, doi:10.1037/1040-3590.7.4.524 (1995).

6 Sardá Junior, J. *et al.* Validation of the Pain-Related Catastrophizing Thoughts Scale. *Acta Fisiátrica* **15**, doi:10.5935/0104-7795.20080001 (2008).

7 Williamson, A. & Hoggart, B. Pain: a review of three commonly used pain rating scales. *J Clin Nurs* **14**, 798-804, doi:10.1111/j.1365-2702.2005.01121.x (2005).

8 Steele, B. Timed walking tests of exercise capacity in chronic cardiopulmonary illness. *J Cardiopulm Rehabil* **16**, 25-33, doi:10.1097/00008483-199601000-00003 (1996).

9 Podsiadlo, D. & Richardson, S. The timed "Up & Go": a test of basic functional mobility for frail elderly persons. *J Am Geriatr Soc* **39**, 142-148, doi:10.1111/j.1532-5415.1991.tb01616.x (1991).

10 Compston, A. Aids to the investigation of peripheral nerve injuries. Medical Research Council: Nerve Injuries Research Committee. His Majesty's Stationery Office: 1942; pp. 48 (iii) and 74 figures and 7 diagrams; with aids to the examination of the peripheral nervous system. By Michael O'Brien for the Guarantors of Brain. Saunders Elsevier: 2010; pp. [8] 64 and 94 Figures. *Brain* **133**, 2838-2844, doi:10.1093/brain/awq270 (2010).

11 Stevenson, T. J. Detecting change in patients with stroke using the Berg Balance Scale. *Aust J Physiother* **47**, 29-38, doi:10.1016/s0004-9514(14)60296-8 (2001).

12 Johns, M. W. Reliability and factor analysis of the Epworth Sleepiness Scale. *Sleep* **15**, 376-381, doi:10.1093/sleep/15.4.376 (1992).

13 Freire, M. Á. *et al.* Escala Hamilton: estudo das características psicométricas em uma amostra do sul do Brasil. *Jornal Brasileiro de Psiquiatria* **63**, 281-289, doi:10.1590/0047-2085000000036 (2014).

14 Botega, N. J., Bio, M. R., Zomignani, M. A., Garcia, C., Jr. & Pereira, W. A. [Mood disorders among inpatients in ambulatory and validation of the anxiety and depression scale HAD]. *Rev Saude Publica* **29**, 355-363, doi:10.1590/s0034-89101995000500004 (1995).

15 Bellamy, N., Buchanan, W. W., Goldsmith, C. H., Campbell, J. & Stitt, L. W. Validation study of WOMAC: a health status instrument for measuring clinically important patient relevant outcomes to antirheumatic drug therapy in patients with osteoarthritis of the hip or knee. *J Rheumatol* **15**, 1833-1840 (1988).

16 Kellgren, J. H. & Lawrence, J. S. Radiological assessment of osteo-arthrosis. *Ann Rheum Dis* **16**, 494-502, doi:10.1136/ard.16.4.494 (1957).
